# Comprehensive Genomic Profiling Reveals the Mutational Spectrum and Clinical Significance of BRCA1/2 and Other Cancer-Susceptibility Genes in Breast Cancer Patients from Southern Tunisia

**DOI:** 10.3390/cancers18172810

**Published:** 2026-08-29

**Authors:** Nihel Ammous-Boukhris, Rania Abdelmaksoud-Dammak, Wala Ben Kridis, Dorra Ben-Ayed-Guerfali, Souhir Guidara, Ameni Feki, Hassen Kamoun, Afef Khanfir, Jamel Daoud, Gérard-Hubert Lizard, Ali Gargouri, Raja Mokdad-Gargouri

**Affiliations:** 1 Center of Biotechnology of Sfax, Laboratory of Molecular Biotechnology of Eukaryotes, University of Sfax, Sfax 3018, Tunisia; ammousnihel@yahoo.fr (N.A.-B.); abdel.rania@gmail.com (R.A.-D.); dorra.benayed@gmail.com (D.B.-A.-G.); faouzi.gargouri@cbs.rnrt.tn (A.G.); 2 Department of Medical Oncology, Habib Bourguiba Hospital, Sfax 3018, Tunisia; walabenkridis@yahoo.fr (W.B.K.); dr.cherif.ameni@gmail.com (A.F.);; 3 Department of Genetics, Hedi Chaker Hospital, Sfax 3018, Tunisia; souhir.guidara89@gmail.com (S.G.);; 4 Department of Radiotherapy, Habib Bourguiba Hospital, Sfax 3018, Tunisia; 5 Laboratoire Interdisciplinaire Carnot de Bourgogne ICB UMR 6303, University of Bourgogne Europe/CNRS/Inserm, 21000 Dijon, France; gerard.lizard@u-bourgogne.fr

**Keywords:** breast cancer, *BRCA1*, *BRCA2*, pathogenic/likely pathogenic variant, variant of unknown significance, targeted next-generation sequencing

## Abstract

Background/Objectives: This study aims to investigate the mutational spectrum of *BRCA1* and *BRCA2* genes in a cohort of breast cancer (BC) patients from southern Tunisia, and to evaluate their clinical and prognostic significance. Additionally, this study explores the contribution of other cancer predisposition genes and the prevalence of variants of uncertain significance (VUS). Results: Among the 165 patients included, pathogenic or likely pathogenic variants (P/LPVs) in *BRCA1/BRCA2* were identified in 19 cases (11.51%), including 8 in *BRCA1* and 11 in *BRCA2*. The presence of *BRCA* P/LPVs associated with young patients (*p* = 0.006) and those with TNBC (*p* = 0.036). Beyond *BRCA1/2*, PV/LPVs were detected in other cancer-related genes, including *TP53* (*n* = 3), *CHEK2*, *RAD50* (*n* = 2 cases each), and *MUTYH*, *BARD1*, and *BRIP1* (one case each). Furthermore, 56 VUS were identified; among them, 7 were prioritized based on in silico predictive analyses, suggesting a potential deleterious effect. However, these VUS should not be used for clinical decision-making without additional evidence from functional and familial segregation studies. Conclusions: Our findings provide novel insights into the genetic landscape of breast cancer in southern Tunisia, highlighting the clinical relevance of *BRCA1/2* mutations and the contribution of other susceptibility genes. These results support the personalized management of breast cancer patients and the implementation of expanded multigene panel testing in routine clinical practice to improve genetic counseling.

## Simple Summary

Breast cancer is one of the most common cancers among women, but its genetic causes remain poorly characterized in North African populations. In this study, we investigated germline genetic variants in 165 Tunisian breast cancer patients using targeted next-generation sequencing of a multigene cancer panel. We identified pathogenic or likely pathogenic variants (P/LPVs) in *BRCA1* and *BRCA2* genes in 19 patients (11.5%), with several recurrent variants and additional variants not previously reported in our Tunisian cohort. P/LPV carriers were more frequently younger at diagnosis and showed significant associations with family history and several clinical features. We also identified P/LPVs in other genes. In addition, 56 variants of uncertain significance (VUS) were detected, of which 7 were prioritized through computational analyses. Additional functional or segregation evidence should be collected to establish pathogenicity. Our findings expand the available genetic data on breast cancer in Tunisia and highlight the importance of population-specific genomic studies for improving variant interpretation and genetic counseling.

## 1. Introduction

Breast cancer (BC) is one of the leading causes of death worldwide in the female population, with an estimated 2,600,000 cases and 685,000 deaths in 2022 [1,2]. In Tunisia, the incidence of BC is approximately 30/100,000 per year [3,4]. Several risk factors, such as hormonal regulation and lifestyle, have been associated with BC development [5,6]; however, heredity remains one of the most significant risk factors [7]. In fact, 5 to 10% of BCs are attributable to hereditary syndromes with autosomal dominant transmission, such as hereditary breast and ovarian cancer syndrome (HBOC). Two major predisposition genes (*BRCA1* and *BRCA2*) are responsible for 30–50% of HBOC cases [8]. *BRCA1* (MIM#113705) and *BRCA2* (MIM# 600185) are tumor suppressor genes that play a crucial role in DNA repair through the homologous recombination pathway [8]. Loss of the BRCA function impairs DNA repair, resulting in high mutation rates and thus contributing to tumor development [9,10].

Indeed, women carrying pathogenic germline mutations in *BRCA1/BRCA2* genes have an increased risk of developing HBOC compared to those with wild-type *BRCA* genes [8,11,12].

Advances in genomics through next-generation sequencing (NGS) have revolutionized cancer diagnosis and management, stratifying risk, guiding surveillance, and informing therapeutic decisions. In BC, sequencing is essential for genetic counseling of at-risk relatives, tailored medical follow-up, and eligibility for PARP inhibitor therapies [3,4].

Thousands of pathogenic and likely pathogenic variants (PV/LPVs) have been identified in the *BRCA1* and *BRCA2* genes since they were first studied [12].

Importantly, the prevalence and spectrum of *BRCA1/2* PVs/LPVs vary considerably between different ethnic groups and geographic regions. Because of this variability, many population-based studies have been conducted to map the mutational profiles of these genes in various countries [13,14].

In the Middle East and North Africa (MENA) region, where BC incidence, age at onset, and clinical presentation often differ from those of Western populations, characterizing the *BRCA1/2* mutational profile is particularly important. Studies conducted in several MENA countries have reported the prevalence of germline *BRCA1/2* PVs/LPVs among BC patients, emphasizing both shared and unique variant patterns compared to other populations [15,16,17,18]. These findings bring attention to the need for region-specific genetic testing panels and personalized clinical management approaches.

Tunisia has a complex population history due to its geographical position at the crossroads of North Africa and the Mediterranean. Its genetic diversity reflects contributions from North African, Mediterranean, Middle Eastern, and sub-Saharan populations. The population also shows varying degrees of endogamy and consanguinity, which may contribute to the persistence of recurrent genetic variants in some communities.

Previous genetic studies have identified both Tunisian-specific and variants shared with other North African and Middle Eastern populations, highlighting the genetic diversity of the region. Therefore, characterizing *BRCA1*/2 variation specifically in Tunisian patients may help identify recurrent or population-associated variants that could be relevant to genetic testing and clinical management in Tunisia [19].

In Tunisia, previous studies have reported that between 14.1% and 37% of BC patients carry pathogenic *BRCA* variants [18,19,20,21]. Several recurrent mutations have been identified within this population, suggesting the existence of population-specific mutational patterns [22]. Identifying these recurrent variants is crucial for the development of targeted genetic screening strategies and for improving clinical management tailored to the Tunisian context.

The primary objective of this study is to expand the mutational spectrum of the *BRCA1* and *BRCA2* genes by identifying germline pathogenic and likely pathogenic variants with or without family history of cancer in patients selected from the south Tunisian region. This effort aims to support therapeutic decision-making and provide more effective genetic counseling for Tunisian BC patients and their families.

## 2. Materials and Methods

### 2.1. Study Population

A cohort of 165 unrelated Tunisian breast cancer patients (162 females and 3 males), enrolled from the Habib Bourguiba Hospital of Sfax-Tunisia’s Medical Oncology Department between 2020 and 2024, was included in this study. All patients underwent germline genetic testing based on clinical features, including early onset of breast cancer (<35 years), bilateral disease, triple-negative phenotype, or family history of breast and/or ovarian cancer. Among these patients, 116 (70.3%) have a familial history of cancer according to the NCCN guidelines [11] and 37/165 (22.4%) were diagnosed with triple-negative breast cancer (TNBC).

Written informed consent was obtained from all participants, and the study was conducted in accordance with the Declaration of Helsinki and approved by the ethics committee of the Faculty of Medicine of Sfax, Tunisia.

### 2.2. DNA Extraction and Targeted Next-Generation Sequencing

Genomic DNA was extracted from peripheral blood using QIAamp Blood DNA (Qiagen, Hilden, Germany), according to the manufacturer’s protocol. Concentration and purity of the DNA were measured using a NanoDrop spectrophotometer and Qubit fluorometer (dsDNA HS kit, Thermo Fisher Waltham, Masachussetts, MA, USA).

DNA libraries were generated using the OncoRisk Cancer Panel (Celemics, Seoul, Republic of Korea) according to the manufacturers’ protocols. This panel includes 31 genes: *APC*, *ATM*, *BARD1*, *BLM*, *BMPR1A*, *BRCA1*, *BRCA2*, *BRIP1*, *CDH1*, *CDK4*, *CDKN2A*, *CHEK2*, *EPCAM*, *MLH1*, *MRE11A*, *MSH2*, *MSH6*, *MUTYH*, *NBN*, *PALB2*, *PMS2*, *PRSS1*, *PTEN*, *RAD50*, *RAD51C*, *RAD51D*, *SLX4*, *SMAD4*, *STK11*, *TP53*, and *VHL*.

Sequencing was performed on an Illumina MiSeq Instrument (Illumina, San Diego, CA, USA) with read lengths of 2 × 151 bp using V3 flow cell (Illumina). The average sequencing depth was 1405.85, with a minimum of 100× for variant calling. Raw reads were analyzed using the basespace cloud-based tool by Illumina.

### 2.3. Variant Classification

Variants were classified according to the American College of Medical Genetics and Genomics (ACMG) guidelines into five categories: pathogenic, likely pathogenic, variant of uncertain significance (VUS), likely benign, and benign. Pathogenicity was assessed after comparing the data with sequence databases: ClinVar (https://www.ncbi.nlm.nih.gov/clinvar/), accessed on 1 April 2026, Varsome (https://varsome.com) and Franklin (Qiagen). Only pathogenic variants, likely pathogenic variants, and VUS are discussed in this study.

### 2.4. In Silico Pathogenicity Prediction

All VUS identified in this study underwent extended bioinformatic characterization to assess their potential clinical relevance. Each VUS was investigated using a panel of computational tools covering different mechanisms: Protein function impact: PolyPhen-2 (probably damaging/benign), SIFT (deleterious/tolerated), Mutation Taster (disease-causing to most sensitive), REVEL, Alpha missense, FATHM, and CADD (Combined Annotation Dependent Depletion); Threshold used: CADD ≥ 20 means potentially pathogenic, in addition to Regulome, SpliceAI, and SPIP, for intronic variants. Variants appearing in two or more unrelated patients were flagged as recurrent VUS, potentially indicating founder effects or population enrichment.

### 2.5. Statistical Analysis

Descriptive statistics were generated using SPSS V.30.0.

The following analyses were performed: frequency of PV/LPV and VUS per gene, distribution by age and clinical parameters, comparison of recurrent vs. non-recurrent VUS. A *p*-value < 0.05 was considered significant. Kaplan–Meier was used to correlate the overall survival in patients carrying or not germline P/LPVs in *BRCA1/2* genes.

## 3. Results

### 3.1. Patient Cohort and Clinical Features

A total of 165 BC patients were enrolled in our study. The median age at diagnosis was 43.9 (range, 24–75 years); most were female (98.1%) and all were from the south region of Tunisia. A family history of breast and/or ovarian cancer was reported in 70.31% of patients. Overall, 92 patients (55.8%) had SBR II invasive ductal carcinoma, 21 patients (12.72%) had bilateral BC and 37 (22.4%) had triple-negative BC. Twenty-five patients (15.15%) died of the disease (Table 1).

### 3.2. Pathogenic/Likely Pathogenic Variants

**Figure 1:**
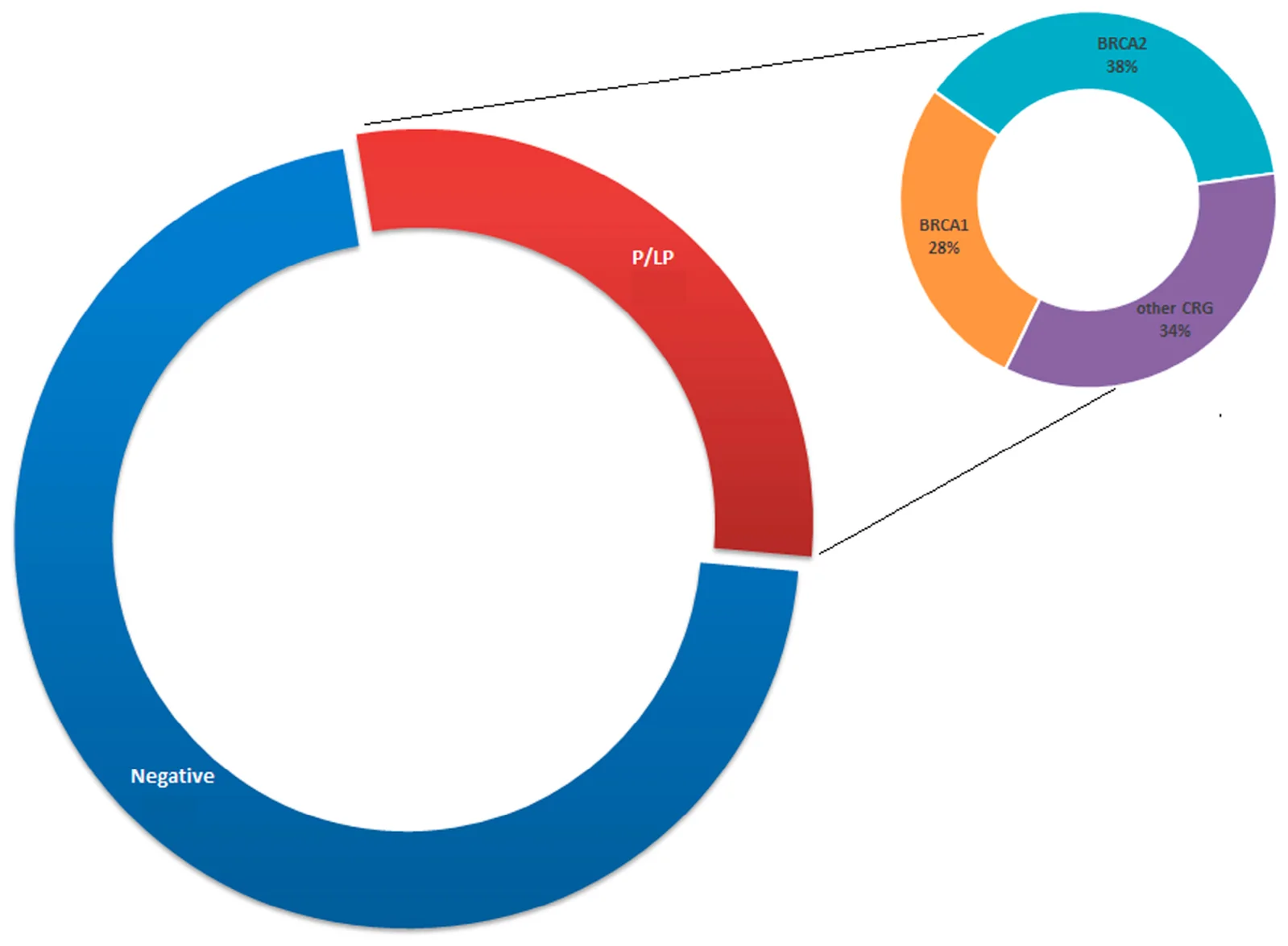
Spectrum of the germline genomic profile in Tunisian BC patients: distribution of all germline variants identified in 165 Tunisian BC patients, classified according to ACMG guidelines.

Among 165 BC patients, 19 (11.51%) carried germline *BRCA1* (n = 8) and *BRCA2* (n = 11) P/LPVs, while 10 patients (6.06%) had P/LPVs in other cancer-related genes such as *RAD50*, *CHEK2*, *TP53*, etc. (Figure 1). Most of these variants had been previously reported in the ClinVar and dbSNP databases, whereas three were novel (Table 2). Recurrent PVs in *BRCA2* (c.8486A>G and c.3248delA) and *BRCA1* (c.1129del) were each identified in two unrelated patients (Table 2). Most P/LPVs were frameshift variants (six in *BRCA1* and five in *BRCA2*), while four were missense, two of which were not previously reported in the ClinVar database (Table 2). Furthermore, one patient carried both a frameshift and splice P/LPV in *BRCA2* (c.1310_1313del./c.632-1G>A). Besides *BRCA1/2*, we identified P/LPVs in *MUTYH*, *RAD50*, *Chek2*, *TP53*, and *BRIP1* genes in 10 patients (Table 3). It is important to mention that *BRCA1*, *BRCA2*, *TP53*, and *CHEK2* were considered in the context of breast cancer susceptibility, whereas variants identified in *BRIP1*, *MUTYH*, *RAD50*, and *APC* were interpreted according to their established or uncertain gene–disease associations and were not considered evidence of breast cancer predisposition in this cohort.

Interestingly, five patients carried concomitant pathogenic variants, including a pathogenic *BRCA2* variant together with an additional pathogenic variant in another cancer-susceptibility gene. Notably, all five patients had a strong family history of breast and/or ovarian cancer and were diagnosed at a relatively young age (35–51 years) (Table 2 and Table 3).

Furthermore, we reviewed previously published Tunisian studies, which revealed a heterogeneous *BRCA1*/2 mutational spectrum, with several recurrent P/LPVs reported across different cohorts. Notably, *BRCA1* c.211dupA and c.5266dupC, BRCA1 c.5030_5033delCTAA, and *BRCA2* c.1310_1313delAAGA have been repeatedly identified in Tunisian families or patients (Table 4).

### 3.3. Association of BRCA P/LPV and Clinico-Pathological Features

**Figure 2:**
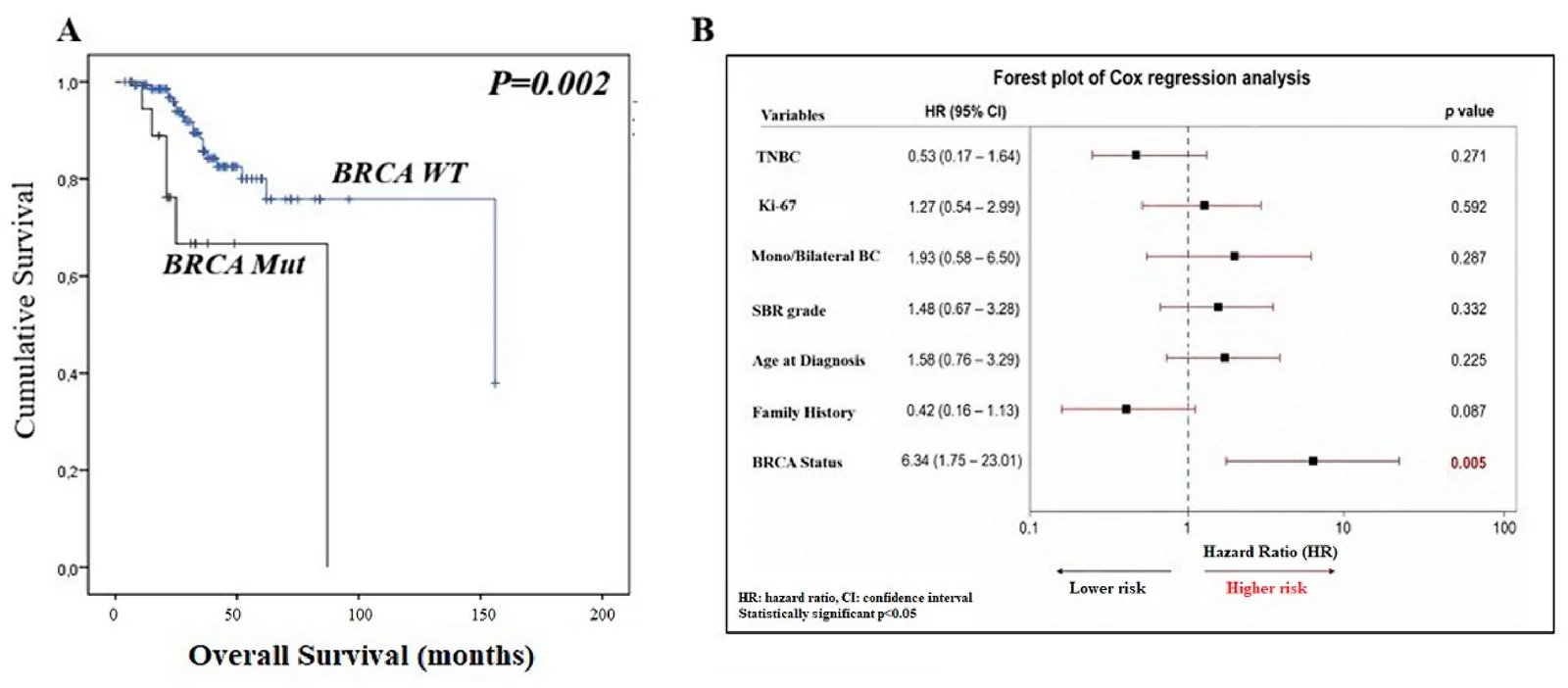
Prognostic impact of BRCA mutation status on overall survivaland Cox regression analysis. (**A**) Kaplan–Meier overall survival curves comparing patients harboring BRCA pathogenic variants (BRCA Mut) with those carrying wild-type BRCA (BRCA WT). (**B**) Forest plot of the univariable Cox proportional hazards regression analysis evaluating the association between clinicopathological variables and overall survival.

*BRCA* status was significantly associated with age at diagnosis, with a higher prevalence of *BRCA* mutations among younger patients (*p* = 0.006, Table 5). In addition, *BRCA* P/LPVs were significantly associated with a positive family history (*p* = 0.042), hormonal status (*p* = 0.036), and presence of metastasis (*p* = 0.048) (Table 5). Survival analysis revealed that carriers of BRCA1/2 pathogenic or likely pathogenic variants (P/LPVs) had significantly shorter overall survival than non-carriers (log-rank *p* = 0.002; Figure 2A). In addition, Cox proportional hazards regression analysis demonstrated that BRCA1/2 mutation status was significantly associated with overall survival (HR = 6.336, 95% CI: 1.745–23.007; *p* = 0.005; Figure 2B).

### 3.4. Variants of Uncertain Significance (VUS)

**Figure 3:**
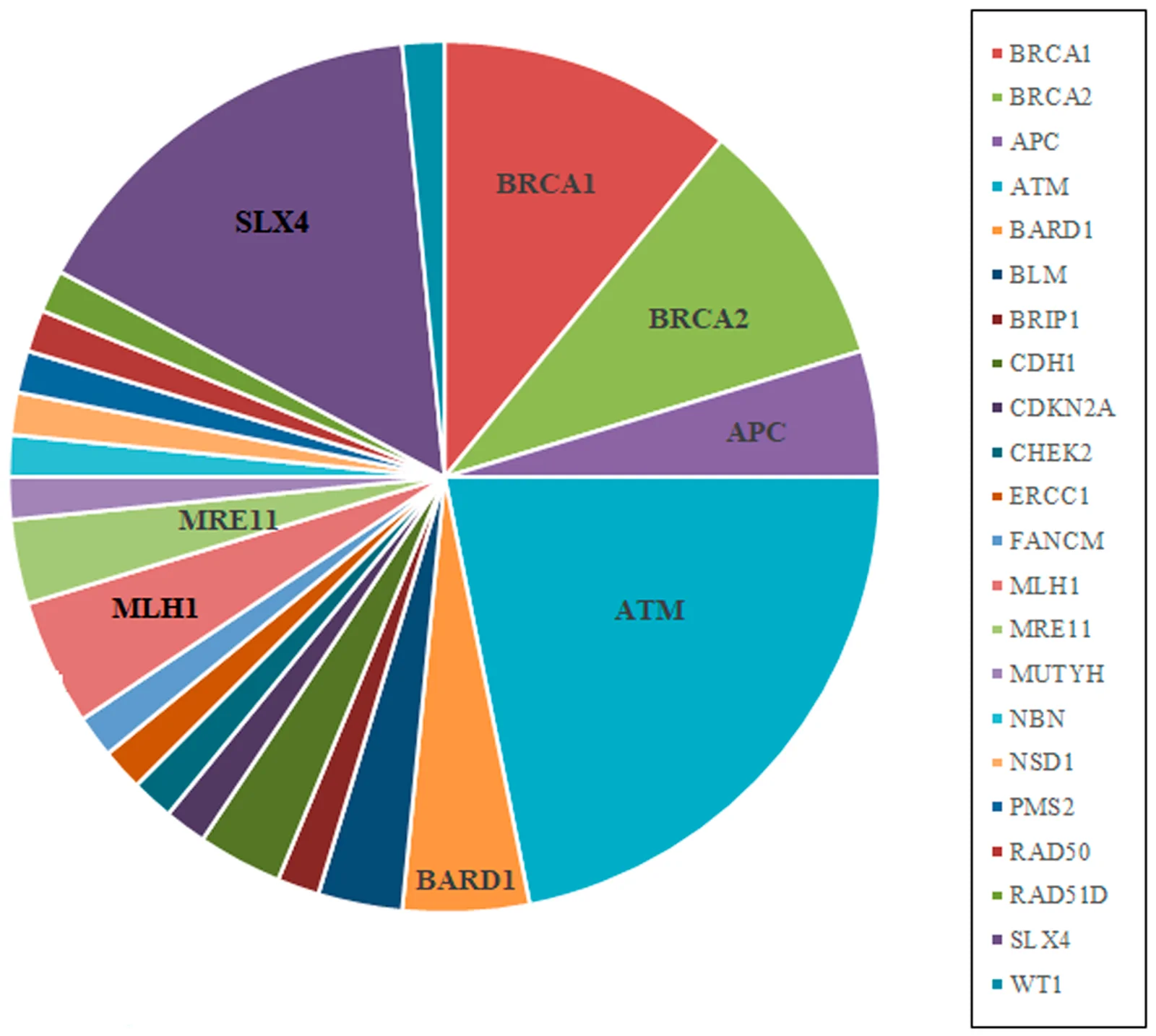
Distribution of variants of uncertain significance (VUS) identified in the study cohort. The pie chart shows the distribution of VUS across the different genes, with the highest frequencies observed in *ATM*, *BRCA2*, and *BRCA1*.

Several variants of uncertain significance (VUS) were identified across the following genes: *ATM* (n = 11), *SLX4* (n = 7), *BRCA2* and *BRCA1* (n = 6 each), *APC*, *BARD1*, *MLH1* (n = 3 each), *CDH1*, *MRE11* (n = 2 each), *BLM*, *BRIP1*, *CDNK2A*, *CHEK2*, *ERCC1*, *FANCM*, *MSH3*, *MSH6*, *MUTYH*, *PMS2*, *NBN*, *RAD50*, *RAD51D*, *NSD1*, and *WT1* (n = 1 each) (Supplementary Table S1). Overall, 64 patients (38.7%) harbored VUS distributed across 25 genes. *ATM* and *SLX4* accounted for the largest number of VUS, with 11 and 7 variants, respectively (Figure 3).

Three recurrent VUS in *ATM* (p.Phe1463Cys, p.Leu1498Ile, and p.Arg2105Gly) were each identified in two unrelated patients. In addition, other recurrent VUS in *BLM* (c.-78del), *MUTYH* (c.583A>G), and *SLX4* (c.-416G>A) were identified in two and four unrelated patients, respectively (Supplementary Table S1).

Co-occurrence of a VUS with a P/LPV in another gene was documented in nine patients. Notably, one patient (334) harbored two PVs in *BRCA2* and *RAD50*, in addition to a VUS in *BLM* (Table 2 and Table 3). This patient has a strong family history of BC and is aged 45 years old.

Among the identified VUS, seven variants were considered of particular interest, being predicted as deleterious by in silico prediction tools and/or expected to affect protein function (Table 6). They include five missense variants localized in *ATM* and *CHEK2* genes, one novel splicing variant in *BRCA2*, and one in-frame delins variant in the *BRCA1* gene (Table 6).

**Figure 4:**
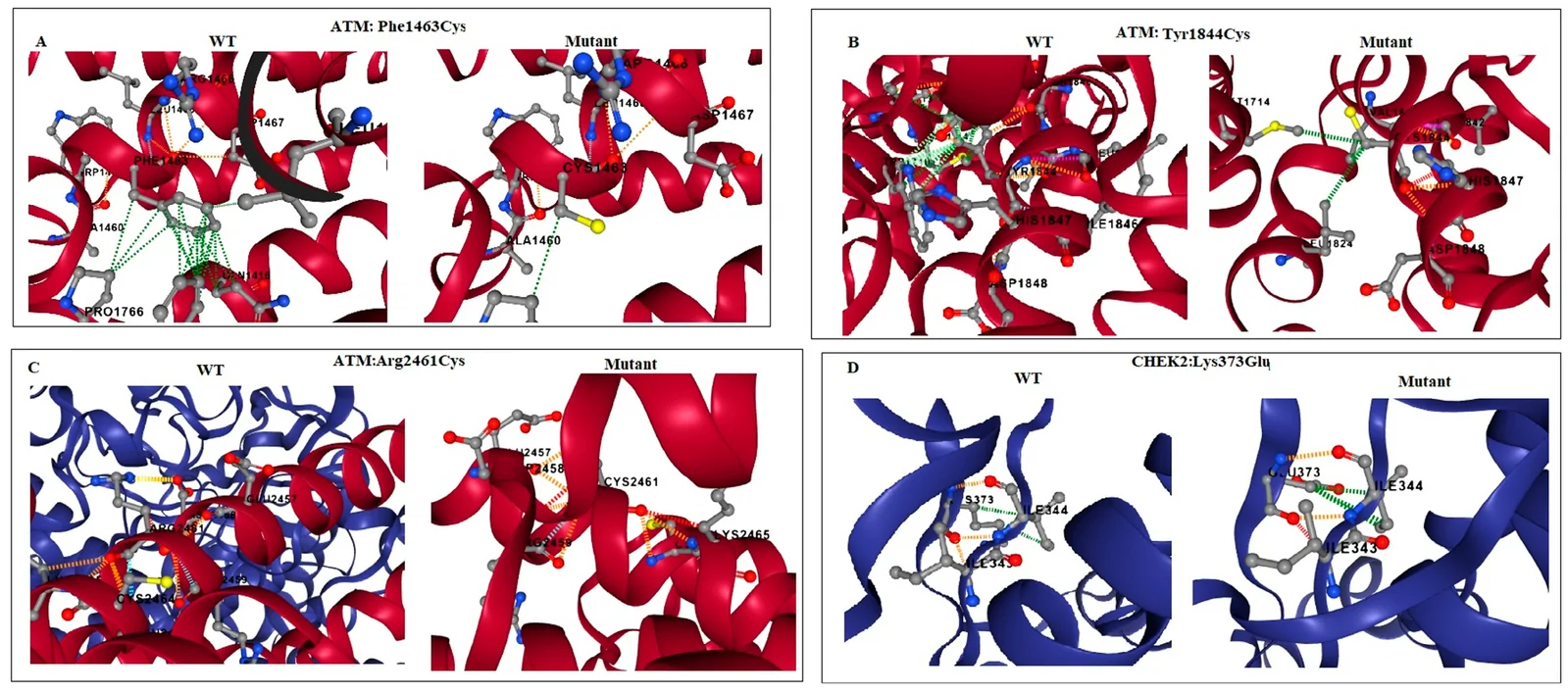
Prediction of the 3D structure of wild-type and mutant proteins of selected VUS. (**A**): Phe1463Cys, (**B**): Tyr1844Cys, (**C**): Arg2105Gly in ATM, and (**D**): Lys373Glu in CHEK2 using DynaMut2. Protein structures are displayed as ribbon representations, while the mutated residues and neighboring amino acids are shown as sticks.

Two *ATM* variants (c.4388T>G and c.6313 A>G) were each found in two unrelated patients with a strong family history of BC. Both variants sit within the regulatory FAT domain of the ATM protein and are likely to destabilize the protein according to their scores in DynaMutΔΔG of −1.62 and −1.02, respectively. Additionally, among the other investigated *ATM* variants, and based on DynaMut predictions, we found that the Tyr1844Cys may lead to protein destabilization while the Arg2461Cys may have a stabilizing effect (Figure 4).

Furthermore, a novel *BRCA2* VUS was identified in a young patient diagnosed at 28 years with a triple-negative breast cancer (TNBC). Splice-site prediction analysis suggested that this variant may substantially impair the donor splice site (reference score: 82.26; mutated score: 71.06; Δ = −13.62%), indicating a potential disruption of the normal mRNA processing and thus the BRCA2 protein function.

Another novel in-frame deletion–insertion (c.2077_2082delGACAGCinsAACAGT) in exon 10 of the *BRCA1* gene was detected in one patient diagnosed with breast and ovarian cancer at the age of 40. This alteration is predicted to remove two amino acids (Ser694 and Asp695) within the DNA-binding domain of BRCA1 protein, which may have strong implications for the structural stability and the function of the protein.

Importantly, these in silico predictions were used solely to prioritize VUS for further investigation and do not establish their pathogenicity or clinical significance. Accordingly, the identified VUS should not be used for clinical decision-making in the absence of additional evidence from functional and familial segregation studies.

## 4. Discussion

In this study, we report the molecular profiling results of 165 Tunisian BC patients using targeted next-generation sequencing (NGS). In total, we identified 29 pathogenic or likely pathogenic variants (P/LPVs) distributed across eight cancer-predisposition genes. As expected, *BRCA1* and *BRCA2* constituted the major altered genes, accounting for 19 out of 29 (65.51%) P/LPVs. The remaining variants were distributed amongst *TP53*, *CHEK2*, *RAD50*, *MUTYH*, *BARD1,* and *BRIP1* genes, reflecting the genetic heterogeneity of BC susceptibility in Tunisia.

It is well documented that the prevalence of *BRCA1*/*BRCA2* P/LPVs in BC varies considerably among populations due to differences in ethnic background, founder effects, selection criteria, and testing strategies [13,14,18]. In North African countries, reported frequencies range between 11% to 37%, highlighting both regional heterogeneity and the under-representation of this population in global genetic studies [15,16,17,18]. In our cohort, 16 *BRCA1*/*BRCA2* PVs were detected in 19 out of 165 patients (11.5%), which aligns with the range of previously reported frequencies for the region [4,22].

Interestingly, three recurrent variants, one in *BRCA1* (c.1129del) and two in *BRCA2* (c.8486A>G and c.3248delA), were each found in two unrelated patients. Other recurrent *BRCA* PVs were previously reported in Tunisian BC patients such as c.5030_5033delCTAA in *BRCA1* and c.1310-1313 delAAGA in *BRCA2* [4]. Their recurrence may suggest local founder effects or population-specific mutational hotspots, consistent with prior reports of geographically restricted *BRCA* variants in North Africa [18]. Interestingly, none of the newly *BRCA1*/2 P/LPVs identified in the present cohort overlapped with those reported in this previous cohort. This finding indicates that the present study expands, rather than duplicates, the previously reported *BRCA1*/2 mutational spectrum in southern Tunisia. The identification of additional variants in independent cohorts highlights the genetic heterogeneity of *BRCA1*/2-associated BC in the Tunisian population and underscores the value of continued accumulation and harmonization of national genomic data. Furthermore, by reviewing the Tunisian literature, we observe a heterogeneous *BRCA1*/2 mutational spectrum, with several recurrent variants that may be particularly relevant for developing a national genetic testing strategy. Notably, *BRCA1* c.211dupA and c.5266dupC, *BRCA1* c.5030_5033delCTAA, and *BRCA2* c.1310_1313delAAGA have been repeatedly identified in Tunisian families or patients, although their relative frequencies may vary by geographical region and patient-selection criteria.

Moreover, three *BRCA1* P/LPVs and two *BRCA2* P/LPVs identified in this study have not been previously reported, thereby expanding the mutational landscape of hereditary BC in Tunisian patients and underlining the value of including under-represented populations in global genetic research.

Furthermore, it is important to mention that a comprehensive Tunisian *BRCA1*/2 variant resource integrating data from different cohorts and institutions would be particularly valuable for establishing variant frequencies, identifying recurrent or population-enriched variants, and supporting evidence-based genetic counseling and testing strategies.

To further contextualize the *BRCA1*/2 mutational spectrum observed in our Tunisian cohort, we compared our findings with data reported from neighboring North African populations, particularly Morocco. The Moroccan study by Elalaoui et al. [15] analyzed 163 unrelated patients with hereditary breast and/or ovarian cancer and identified a distinct spectrum of recurrent *BRCA1*/2 variants, with *BRCA2* c.1310_1313delAAGA representing the most frequent variant in their cohort. In our cohort, the same *BRCA2* c.1310_1313delAAGA variant was identified in one patient, indicating that some PVs are shared across North African populations. In addition, *BRCA1* c.5266dupC, identified in our cohort, has previously been reported among Moroccan patients. Conversely, several P/LPVs identified in our cohort were not among the recurrent variants highlighted in the Moroccan cohort, supporting the presence of substantial heterogeneity in the *BRCA1*/2 mutational spectrum across North African populations.

In our cohort, family history proved to be a strong predictor of *BRCA1*/*BRCA2* PV status. Patients carrying *BRCA1*/2 P/LPVs were significantly more likely to report a family history of breast or ovarian cancer, a finding that is consistent with previously studies [24,25]. Moreover, 27% of TNBC patients are *BRCA* positive, which is in line with reported data [4,17,26].

Beyond *BRCA1*/2, P/LPVs were identified in several additional cancer-related genes, including *TP53*, *CHEK2*, *RAD50*, *MUTYH*, *APC*, and *BRIP1*. However, these findings should not be interpreted uniformly as breast cancer-susceptibility variants. *TP53* and *CHEK2* have established relevance to hereditary breast cancer risk, whereas the clinical relevance of heterozygous variants in *BRIP1*, *MUTYH*, and *RAD50* to breast cancer predisposition is not established. In particular, *BRIP1* is an established ovarian cancer-susceptibility gene, while heterozygous *MUTYH* variants are primarily associated with carrier status for *MUTYH*-associated polyposis rather than autosomal-dominant hereditary BC. Accordingly, these findings were considered as additional germline variants identified through multigene testing rather than as evidence of non-*BRCA* hereditary BC predisposition [25,27].

In our cohort, four patients presented the co-occurrence of multiple P/LPVs, and one of them harbored two PVs located in *BRCA2* and *RAD50*, together with a VUS located in the *BLM* gene. This patient had a strong family history and developed BC at an early age. Such findings highlight the complexity of hereditary BC genetics and emphasize the need for careful clinical interpretation when multiple variants are identified.

A substantial number of variants of uncertain significance (VUS) were detected (n = 56), particularly in *ATM*, *SLX4*, *BRCA1*, *BRCA2*, and *APC* genes. Based on prediction tools, seven relevant VUS in *ATM*, *CHEK2*, *BRCA1* and *BRCA2* were retained. Interestingly, two *ATM* VUS were each detected in two unrelated patients. These variants are located within the FAT domain and are predicted to affect protein stability and potentially impair ATM protein function. Nevertheless, additional investigations such as familial segregation studies are required to clarify their pathogenicity and to determine whether they are associated with BC risk. We also identified two novel VUS: a frameshift deletion in the *BRCA1* gene and a splice-site variant in the *BRCA2* gene, both of which were predicted to affect the function of the corresponding proteins. The *BRCA1* VUS is characterized by the deletion of two amino acids (Ser694 and Asp695) within the DNA-binding domain, potentially altering the structural integrity of the protein. The *BRCA2* variant is predicted to disrupt the donor splice site, and further RNA-based analyses are needed to confirm whether it induces aberrant splicing, including potential exon skipping. Numerous studies have concentrated on the reclassification of VUS, especially those occurring in *BRCA* and *ATM* genes, which are among the most frequently implicated in hereditary BC [28,29].

Recently, a study of Tunisian BC patients reported the reclassification of two VUS located in *ATM* (c.6115G>A), and *CHEK2* (c.592+3A>T) genes, into likely pathogenic variants using a combination of familial segregation analysis and comprehensive in silico prediction tools [30].

While expected in multigene panel studies, the high VUS rate reflects both the genetic heterogeneity of the Tunisian population and the current limitations of international databases, which under-represent North African genomic information, and thus highlights the urgent need for population-specific functional and segregation analyses to clarify its clinical significance, especially in genetically understudied populations such as Tunisia.

This study has some limitations. The sample size may not fully represent the genetic diversity of the Tunisian population. The gene panel used was limited and might have missed some relevant pathogenic variants. Additionally, the identified VUS, and especially the relevant VUS, need further functional validation to clarify their clinical significance. Future studies with larger cohorts and expanded panels are needed.

## 5. Conclusions

This study provides a comprehensive molecular characterization of Tunisian BC patients using NGS multigene panel testing. By identifying pathogenic and likely pathogenic variants in individuals at high hereditary risk, and by thoroughly annotating and prioritizing variants of uncertain significance (VUS), we reveal both recurrent and novel mutations that point to population-specific genomic patterns. The integration of NGS with multiple in silico prediction tools enabled the prioritization of VUS for further investigation; however, these computational predictions do not establish pathogenicity or clinical significance.

Through the generation of such locally relevant genomic data, this work moves Tunisia closer to the implementation of effective precision medicine strategies, where cancer management is adapted to the genetic landscape of the Tunisian population.

## Figures and Tables

**Table 1:** Clinical characteristics of BC patients. Correlation of clinical features with mutational status. TNBC: triple-negative breast cancer.

Characteristics	N (%)
Age (y)	
≤35	39 (23.6)
36–55	107(64.84)
>55	19 (11.51)
Hormonal status	
Luminal A	92 (55.8)
Luminal B	28 (17)
TNBC	37 (22.4)
Her2+	8 (4.8)
Family history	
Negative	49 (29.69)
Positive	116 (70.31)
Tumor Site	
Unilateral	144 (87.28)
Bilateral	21 (12.72)
SBR Grade	
I	10 (6.1)
II	92 (55.8)
III	63 (38.2)
Metastasis	
Absence	116 (70.3)
Presence	49 (29.7)
Ki67 index	
>20%	92 (55.8)
≤20%	73 (44.2)
Prognosis	
Dead	25 (15.2)
Alive	140 (84.8)

**Table 2:** *BRCA1/2* PVs/LPVs in 165 BC patients. List of pathogenic/likely pathogenic variants detected in *BRCA1/2* genes.

Patient ID	Variant Location	HGVS Nomenclature	Age	Family History of Cancer	dbSNP rs	Reported
*BRCA1* (NM_007294.4)						
204	Exon10	c.1129del. p.(Ser377Ala fsTer17)	32	Yes Yes	rs2053988613	Yes
316			33			
208	Exon10	c.3968-3971del. p.(Gln1323Arg fsTer12)	41	Yes	rs886040181	Yes
259	Exon 13	c.4484G>A, p.(Arg1495Lys)	47	Yes	rs80357389	Yes
336	Exon 16	c.5030_5033del. p.(Thr1677IlefsTer2)	31	Yes	rs80357580	Yes
350	Exon 5	c.248_249insGAGGA, p.(Glu84fs)	52	Yes		No
367	Exon 19	c.5266dupC, p.Gln1756fs	31	Yes	rs80357906	Yes
330	Exon 10	c.4067_4071del p.(Gln1356ArgfsTer10)	45	Yes	rs2154266021	Yes
*BRCA2* (NM_000059.4)						
289	Exon 19	c.8486A>G, p.(Gln2829 Arg)	51	Yes	rs80359100	Yes
224			40			
255 196	Exon 11	c.3248del. p.(Asn1083IlefsTer4)	46	Yes	rs886040466	Yes
341	Exon 3	c.289G>T, p. (Glu97Ter)	41	Yes	rs397507646	Yes
363	Exon 3	c.315-316del. p.(Gly106fs)	39	No		
327	Exon 11	c.5073dup. p.(Trp1692MetfsTer3)	31	Yes	rs80359479	Yes
328	Exon 11	c.2064T>A, p.(Tyr688 Ter)	48	Yes	-	No
334	Exon 10	c.1813del. p.(Ile605TyrfsTer9)	45	Yes	rs80359306	Yes
384	Exon 10	c.1310_1313del./c.632-1G>A p.Lys437IlefsTer22	67	Yes	rs80359277	Yes
399	Exon 15	c.7533T>G, p.(Tyr2511 Ter)	41	Yes	-	Yes

**Table 3:** P/LPVs in other cancer-related genes in BC patients. List of pathogenic/likely pathogenic variants detected in other cancer-related genes.

Patient ID	Gene	Variant	Age	dbSNP
292	MUTYH NM_001128425.1	c.1227_1228dup p.Glu410GlyfsTer43	45	rs587780078
334	*RAD50* NM_005732.4	c.2801del p.Asn934IlefsTerTer6	45	rs748536322
325	*RAD50* NM_005732.4	c.2801dup p.Asn934Lysfs10	43	rs748536322
289	*BARD1*, NM_000465.4	c.16C>T p.Gln6Ter	51	-
421	*CHEK2,* NM_007194.4	c.1427C>T p.Thr476Met	43	rs142763740
196	*CHEK2* NM_007194.3	c.1260C>A p.Cys420Ter	32	rs762205611
290	*TP53,* NM_000546.6	c.792_794del p.Leu265del	29	
285	*TP53* NM_000546.6	c.497C>G p.Ser166Ter	55	
341	*TP53* NM_000546.6	c.613T>G p.Tyr205Asp	68	rs1057520008
328	*BRIP1* NM_032043.3	c.585del p.Asn196ThrfsTer7	48	

**Table 4:** Reported Tunisian P/LP variants in the literature.

Gene	HGVS c. Variant	Cancer Phenotype	Reported in Tunisian Studies/Population	References
*BRCA1*	c.798_799delTT	BC	High-risk families from central Tunisia	[3]
*BRCA1*	c.212 + 2insG	BC	High-risk families from central Tunisia	[3]
*BRCA1*	c.211dupA	BC	Recurrent; reported in several Tunisian cohorts	[21,23]
*BRCA1*	c.3331_3334delCAAG	BC	High-risk families from central Tunisia	[3]
*BRCA1*	c.1504_1508delTTAAA	BOC families	Familial/early-onset Tunisian cases	[21]
*BRCA1*	c.1612C>T	BOC	Reported in Tunisian carriers	[10]
*BRCA1*	c.2418dupA	BC	Tunisian patients	[10]
*BRCA1*	c.2433delC	BC	Tunisian patients	[10]
*BRCA1*	c.3751dup	BOC	Tunisian hereditary cancer families	[23]
*BRCA1*	c.4041_4042del	BOC	Tunisian hereditary cancer families	[23]
*BRCA1*	c.4067_4071delAAGAA	BOC	Southern Tunisia	[4]
*BRCA1*	c.5266dupC	BOC	Recurrent, reported across multiple Tunisian regions	[3,21,23]
*BRCA1*	c.5030_5033delCTAA	BOC	Highly recurrent in southern Tunisia; detected in both BC and OC patients	[4]
*BRCA2*	c.1310_1313delAAGA	BOC	Recurrent Tunisian mutation, particularly reported in northern and southern cohorts	[4,23]
*BRCA2*	c.1313dupT	Familial/early-onset BOC	Novel mutation initially reported in Tunisian families	[21]
*BRCA2*	c.7654dupT	Familial/early-onset BOC	Novel mutation initially reported in Tunisian families	[21]
*BRCA2*	c.17_20delAAGA	BOC	Southern Tunisia	[4]
*BRCA2*	c.1796_1800delCTTAT	BOC	Southern Tunisia	[4]

**Table 5:** Association of BRCA status with clinicopathological parameters.

Patient Characteristics	BRCA Wild-Type	BRCA1 Mutant	BRCA2 Mutant	*p*-Value
Age (years)				
≤35 (39)	28 (71.8)	5 (12.8)	6 (15.4)	0.006
36–55 (107)	100 (93.5)	3 (2.8)	4 (3.7)	
>55 (19)	18 (94.7)	0 (0)	1 (5.3)	
Hormonal status				
Luminal A	85 (58.2)	3 (37.5)	4 (6.5)	0.036
Luminal B	27 (18.5)	0 (0)	1 (9.1)	
TNBC	27 (18.5)	5 (62.5)	5 (45.5)	
Her2+	7 (4.8)	0 (0)	1 (9.1)	
Family history				
Negative (49)	48 (98)	0 (0)	1 (2)	0.042
Positive (116)	98 (84.5)	8 (6.9)	10 (8.6)	
Tumor Site				
Unilateral (144)	129(89.6)	7 (4.9)	8 (5.6)	0.325
Bilateral (21)	17 (81)	1 (4.8)	3 (14.3)	
Grade				
I (10)	10 (100)	0 (0)	0 (0)	0.062
II (92)	86 (93.5%)	2 (2.2)	4 (4.3)	
III (63)	50 (79.4%)	6 (9.5)	7 (11.1)	
Metastasis				
Absence	107 (92.2)	3 (2.6)	6 (5.2)	0.048
Presence	39 (79.6)	5 (10.2)	5 (10.2)	
Ki67 index				
≥20% (92)	88 (95.7)	2 (2.2)	2 (2.2)	0.005
<20% (73)	58 (79.5)	6 (8.2)	9 (12.3)	

**Table 6:** List of relevant VUS.

Patient	Gene	NM	rs	cDNA Change	Exon	Protein Change	Variant Type	ClinVar	gnomAD Exome Frequency	SIFT	PolyPhen-2	Mutation Taster	CADD Score	Alpha Missense	Revel	FATHMM	Regulome db	Splice AI Lookup	SPIP	DynaMut ∆∆G
341 & 268	*ATM*	NM_000051.3	rs138327406	c.4388T>G	exon 29	p.Phe1463Cys	Missense	Conflicting	0.0016	D	PD	D	26.8	LP (0.83)	D (0.758)	D (0,88)	-	-	-	−1.62 Destab
327 & 267	*ATM*	NM_000051.4	rs879253983	c.6313A>G	exon 43	p.Arg2105Gly	Missense	VUS	0.00000397	D	PD	D	24.8	LP (0.68)	D (0779)	D (0.759)	-	-	-	−1.02 Destab
285	*ATM*	NM_000051.3	rs201314561	c.7381C>T	exon 50	p.Arg2461Cys	Missense	Conflicting	0.0000835	D	PD	D	29.4	LB (0.72)	D (0.707)	D (0.142)	-	-	-	0.536 Stab
331	*ATM*	NM_000051.3	rs876658907	c.5531A>G	exon 37	p.Tyr1844Cys	Missense	VUS	-	D	PD	B	24.3	LB (0.08)	D (0.67)	D (0.523)	-	-	-	−1.73 Destab
300	*CHEK2*	NM_007194.3	rs74751600	c.1117A>G	exon 11	p.Lys373Glu	Missense	VUS	0.00040	D	PD	D	27.7	LP (0.96)	D (0.369)	D (0.727)	-	-	-	0.722 Stab
364	*BRCA1*	NM_007294.4	_	c.2077-2082 delins	exon 10	p.Ser694_Asp695del	In-frame deletion	VUS	-	-	-	-	-	-	-	-	-	-	-	-
318	*BRCA2*	NM_000059.3	rs876659568	c.1909+3A>T	intronic	_	splicing	VUS	-	-	-	-	11.2	-	-	-	5	Donor Loss	Alteration of the consensus splice site	-

## Data Availability

VCF files are available from the corresponding author upon reasonable request. The data of the current study are involved in the article. The patients’ clinicopathological data are presented in Table 1; the detected SNPs and their accession numbers (rs) are illustrated in Table 2 and Table 3 in the manuscript.

## Supplementary Materials

The following supporting information can be downloaded at: https://www.mdpi.com/article/10.3390/cancers18172810/s1, Supplementary Table S1: In silico parameters of variants of uncertain significance identified in the study cohort. List of VUS with in silico predictive scores: comparison of deleteriousness predictions from multiple in silico tools (PolyPhen-2, SIFT, CADD, Mutation Taster, REVEL, Alpha missense, FATHM, SpliceAI, SPIP, Regulome) for all the identified VUS. D: Damaging, PD: Probably Damaging, LB: Likely Benign, D*: Deleterious, N: Neutral, P: Pathogenic, LP: Likely Pathogenic.

## Abbreviations

ACS	American Cancer Society
BC	Breast cancer
NCCN	National Comprehensive Cancer Network
AJCC	American Joint Committee on Cancer
ACMG	American College of Medical Genetics
TNBC	Triple-negative breast cancer
PVs/LPVs	Pathogenic variants/likely pathogenic variants
HGVS	Human Genome Variation Society
MENA	Middle East and North Africa
HER2	Human epidermal growth factor receptor 2
HBC	Hereditary breast cancer
NGS	Next-generation sequencing
PARP	poly ADP-ribose polymerase
