# Supplementary material for: Comprehensive Genomic Profiling Reveals the Mutational Spectrum and Clinical Significance of BRCA1/2 and Other Cancer-Susceptibility Genes in Breast Cancer Patients from Southern Tunisia
Source: Cancers (Basel). 2026 Aug 29;18(17):2810. doi: 10.3390/cancers18172810 (PMC13565643; doi:10.3390/cancers18172810)
Supplement: Supplementary file 1 [file cancers-18-02810-s001.zip › cancers-4458592-supplementary.pdf]

Supplementary Table S1: In silico parameters of variants of uncertain significance identified in the study cohort.

Patient #	Gene	Chromosome Position	Selected variant	rs	Protein Change	Exon	Variant Type	Internal frequency	ClinVar Classification	gnomAD Exome Frequency	SIFT	PolyPhen-2	Mutation Taster	CADD Score	FATHMM	Regulome db	SpliceAI Lookup	SPIP
patient 1	*APC*	Chr5:112173708	c.2417A>G	rs1561576972	p.His806Arg	Exon 16	Missense	1	VUS	not found	T	PD	D	24,1	D			
patient 2	*APC*	Chr5:112176006	c.4715T>C	rs863224543	p.Ile1572Thr	Exon 16	Missense	1	VUS	0,0000119	T	B	D	19,73	N			
patient 3	*APC*	Chr5:112102067	c.3G>A	rs980662480	p.Met1Ile	Exon1	Start-loss	1	VUS	not found	T	B	D	23,8	N			
patient 4	*ATM*	Chr11:108172382	c.5185G>C	rs3092907	p.Val1729Leu	Exon 35	Missense	1	Conflicting	0,0000995	D	B	D	19,8	N			
patient 5 & 6	*ATM*	Chr11:108160480	c.4388T>G	rs138327406	p.Phe1463Cys	Exon: 29	Missense	2	Conflicting	0,0016	D	PD	D	26,8	D			
patient 7 & 8	*ATM*	Chr11:108188214	c.6313A>G	rs879253983	p.Arg2105Gly	Exon 43	Missense	2	VUS	0,00000397	D	PD	D	24,8	D			
patient 9 & 10	*ATM*	Chr11:108163401	c.4492T>A	rs748949478	p.Leu1498Ile	Exon 30	Missense	3	VUS	not found	D	PD	B	18,6	N			
patient 11	*ATM*	Chr11:108121444	c.1252C>A	rs2135316180	p.Gln418Lys	Exon 10	Missense	1	VUS	not found	T	B	D	19,67	N			
patient 12	*ATM*	Chr11:108138010	c.2579A>T	rs761251711	p.Asp860Val	Exon17	Missense	1	VUS	0.00000397	D	B	B	15,14	N			
patient 13	*ATM*	Chr11:108201014	c.7381C>T	rs201314561	p.Arg2461Cys	Exon 50	Missense	2	Conflicting	0.0000835	D	PD	D	29,4	D			
patient 14	*ATM*	Chr11:108124704	c.2062G>A	rs769338089	p.Glu688Lys	Exon: 13	Missense	1	VUS	0.0000198	T	B	D	37	N			
patient 15	*ATM*	Chr11:108142012	c.2956G>A	rs771065430	p.Val986Ile	Exon 20	Missense	1	no data	0.00000398	T	B	D	22,3	N			
patient 16	*ATM*	Chr11:108175436	c.5531A>G	rs876658907	p.Tyr1844Cys	Exon 37	Missense	1	VUS	not found	D	PD	B	24,3	D			
patient 17	*ATM*	Chr11:108183164	c.5945A>T	rs543980602	p.Gln1982Leu	Exon40	Missense	1	no data	not found	D	PD	D	25,1	D			
patient 18	*BARD1*	Chr2:215674166	c.128G>A	rs1060501305	p.Arg43His	Exon1	Missense	1	VUS	not found	D	PD	B	23,7	N			
patient 19	*BARD1*	chr2:215645570	c.1028C>T	rs201032007	p.Thr343Ile	Exon 4	Missense	1	Conflicting	0.000171	T	B	D	15,61	N			
patient 20	*BARD1*	Chr2:215674278	c.16C>T	rs876658234	p.Gln6*	Exon1	Missense	1	Conflicting	not found	n/a	n/a	D	34	n/a			

patient 21 &22	*BLM*	Chr15:91303325	c.-78del	rs760264310	UTR5'	intron 5	intronic	8	VUS	0,0000273	n/a	n/a	not found	0.225	n/a	6	No effect on splicing	No effect on splicing
patient 23	*BRCA1*	Chr17:41277324	c.-56G>A	_	UTR5'	Exon 1	intronic	1	VUS	0.00000657	n/a	n/a	not found	8,161	n/a	_	No effect on splicing	No effect on splicing
patient 24	*BRCA1*	Chr17:41244121	c.3427T>A	_	p.Ser1143Thr	Exon10	Missense	1	no data	not found	T	B	B	13,79	N			
patient 25	*BRCA1*	Chr17:41228525	c.4464T>A	_	p.Asn1488Lys	exon 13	Missense	1	no data	not found	D	PD	B	17,85	N			
patient 26	*BRCA1*	chr17:41243961	c.3587C>T	rs80356944	p.Thr1196Ile	Exon10	Missense	1	Conflicting	0.00000398	D	B	B	7,33	N			
patient 27	*BRCA1*	chr17:43093454	c.2077-2082 del GACAGCINSAACAGT	_	p.Ser694_Asp695del	Exon11	In-frame deletion	1	VUS	not found	n/a	n/a	not found	n/a	n/a			
patient 28	*BRCA1*		c.4357+22C>T	rs1407411387		intron 12	intronic	1	VUS	0,0000039	n/a	n/a	D	3,18	n/a	5	No effect on splicing	No effect on splicing
patient 29	*BRCA2*	chr13:32893217	c.71T>C	rs397507902	p.Leu24Ser	Exon 3	Missense	1	no data	not found	D	PD	D	23,5	N			
patient 30	*BRCA2*	chr13:32906957	c.1342C>T	rs80358422	p.Arg448Cys	Exon10	Missense	1	Conflicting	0.0000123	D	B	B	10,54	N			
patient 31	*BRCA2*	chr13:32906627	c.1012G>A	rs80358396	p.Ala338Thr	exon10	Missense	1	Conflicting	0.000011	T	B	B	1,21	N			
patient 32	*BRCA2*	chr13:32907527	c.1909+3A>T	rs876659568	UTR 5'	Exon10	splicing	1	VUS	not found	n/a	n/a	not found	11,2	n/a	5	Donor Loss	Alteration of the consensus splice site
patient 33	*BRCA2*	chr13:32893412	c.266C>T	rs748609599	p.Pro89Leu	Exon3	Missense	2	VUS	0,0000159	D	PD	D	14,74	N			
patient 34	*BRCA2*	chr13:32911418_32911419	c.2926_2927delinsAT	rs276174831	p.Ser976Ile	Exon11	Missense	1	Conflicting	not found	D	B	D	n/a	n/a			
patient 35	*BRIP1*	chr17:59857660	c.1897A>C	rs765314472	p.I633L	Exon 13	Missense	1	Conflicting	0.0000357	T	PD	D	20,2	D			
patient 36	*CDH1*	chr16:68771362_68771364	c.44_46del	rs587782476	p.Leu15del	EXON 1	In-frame deletion	1	Conflicting	0.0002	n/a	n/a	not found	16,18	n/a			

patient 37	*CDH1*		c.32_46dup	_	p.Leu11_Leu15dup	Exon1		1	VUS	not found	n/a	n/a	not found	n/a	n/a			
patient 38	*CDKN2A*		c.86G>T	_	p.Arg29Leu	Exon 1	Missense	1	VUS	not found	D	PD	D	22,7	N			
patient 39	*CHEK2*	chr22:29091840	c.1117A>G	rs74751600	p.Lys373Glu	Exon11	Missense	1	VUS	0,0004	D	PD	D	27,7	D			
patient 40	*ERCC1*	chr19:45916979	c799T>C	rs146256515	p.Ser267Pro	Exon 9	Missense	1	VUS	0.0002	D	PD	D	23,3	D			
patient 41	*FANCM*	chr14:45624660	c.1394A>G	rs747572587	p.Asn465Ser	Exon8	Missense	1	VUS	0.0000131	T	B	D	18,9	N			
patient 42	*MLH1*	Chr3:37061860	c.944A>G	_	p.His315Arg	Exon11	Missense	1	no data	not found	T	B	D	22,5	D			
patient 43	*MLH1*	chr3:37042515	c.277A>G	rs41295282	p.Ser93Gly	Exon: 3	Missense	1	Conflicting	0.0000159	D	B	D	17,04	N			
patient 44	*MLH1*	Chr3:37067316	c.1227G>C	rs775776362	p.Gln409His	Exon 12	Missense	1	Conflicting	0.0000329	T	B	D	18,78	D			
patient 45	*MRE 11*	chr5:112175211	c.3920T>A	rs61749249	p.Ile1307Lys	Exon16	Missense	1	VUS	0.002	T	P	B	12,05	n			
patient 46	*MRE11*	chr11:94153339_94153347	c.2077_2085dup	_	p.D693_D695dup	Exon20		1	VUS	not found	n/a	n/a	not found	n/a	n/a			
patient 47	*MUTYH*		c.1226G>A	rs587782120	p.R409Q	Exon 13	Missense	1	Conflicting	7.956e-06	T	B	D	3.98	N			
patient 48	*NBN*	chr8:90955569	c.2096T>C	rs1810039016	p.Leu699Pro	Exon 14	Missense	1	VUS	not found	D	PD	D	24.40	D			
patient 49	*NSD1*	chr5:176721528	c.7159C>A	rs1416255762	p.P2387T	Exon 23	Missense	1	VUS	not found	T	B	B	12.14	N			
patient 50	*PMS2*	chr7:6013060	c.2559C>G	rs371673459	p.Ile853Met	Exon15	Missense	1	Conflicting	0,0001	D	PD	D	0.11	N			
patient 51	*RAD50*		c.3835C>T	rs786201810	p.Arg1279Cys	Exon25	Missense	1	VUS	0,00000795	n/a	PD	D	34	D			
patient 52	*RAD51D*	chr17:33446800	c.-168C>T	rs1190807185	UTR 5' Exon 1		intronic	1	VUS	0.00000657	n/a	n/a	B	8.44		4	No effect on splicing	No effect on splicing
patient 53	*SLX4*		c.2830C>A	_	p.Pro944Thr	Exon 12	Missense	1	no data		T	PD	B	2.87	N			
patient 54, 55, 56, 57	*SLX4*		c.-416G>A	rs528320603	UTR5'	Exon 2	intronic	4	VUS	0.000256	n/a	n/a	B	2,66	N	5	No effect on splicing	No effect on splicing
patient 58	*SLX4*		c.1334G>A	rs2151131780	p.Ser445Asn	Exon 6	Missense	1	VUS	not found	T	B	B	0.67	N			
patient 59	*SLX4*		c.4213G>A	rs2151121826	p.Ala1405Thr	Exon 12	Missense	1	VUS	not found	D	PD	D	16.18	N			
patient 60	*SLX4*		c.1672C>T	rs372264472	p.Arg558Trp	Exon 7	Missense	1	VUS	6.422e-05	T	B	B	8.61	N			

patient 61	*SLX4*		c.4464G>C	rs2040543369	p.Leu1488Phe	Exon12	Missense	3	VUS	not found	D	B		1.14	N			
patient 62	*SLX4*		c.421G>T	rs137976282	p.Gly141Trp	Exon2	Missense	2	VUS	0.0008	D	PD	B	18.36	N			
patient 63	*WT1*		c.314C>G	rs948061247	p.Ala105Gly	Exon1	Missense	1	Conflicting	0.0000724	T	n/a	D	0,1317				

Footnotes:

Patient #, anonymized patient number; Gene, official gene symbol; cDNA, coding DNA sequence; rs, Reference SNP identifier; Exon, exon number according to the reference transcript; ClinVar, NCBI Clinical Variation database; gnomAD, Genome Aggregation Database; Internal Frequency: Frequency defined as the proportion of patients carrying the variant in the present cohort; SIFT, Sorting Intolerant From Tolerant; PolyPhen-2, Polymorphism Phenotyping v2; CADD, Combined Annotation Dependent Depletion; FATHMM, Functional Analysis through Hidden Markov Models; RegulomeDB, regulatory annotation database; SpliceAI, splice-altering prediction tool; SPIP, Splicing Prediction.

In silico prediction scores: SIFT: T, tolerated; D, deleterious. PolyPhen-2: B, benign; PD, probably damaging. MutationTaster: D, disease-causing; B, benign. FATHMM: D, damaging; N, neutral. CADD scores ≥20 were considered indicative of potentially deleterious variants. For SpliceAI, higher delta scores indicate a greater predicted probability of splice alteration.
